# Optimizing Partner Notification Programs for Men Who Have Sex with Men: Factorial Survey Results from South China

**DOI:** 10.1371/journal.pone.0157749

**Published:** 2016-07-27

**Authors:** Alberta L. Wang, Rui-Rui Peng, Joseph D. Tucker, Hrishikesh Chakraborty, Myron S. Cohen, Xiang-Sheng Chen

**Affiliations:** 1 Division of Rheumatology, Immunology and Allergy, Brigham and Women’s Hospital, Harvard Medical School, Boston, Massachusetts, United States of America; 2 Shanghai Skin Disease Hospital, Shanghai, China; 3 Institute for Global Health and Infectious Diseases, University of North Carolina at Chapel Hill, Chapel Hill, North Carolina, United States of America; 4 Department of Epidemiology and Biostatistics, University of South Carolina, Columbia, South Carolina, United States of America; 5 National Center for Sexually Transmitted Disease Control, Chinese Academy of Medical Science and Peking Union Medical College Institute of Dermatology, Nanjing, China; Public Health Agency of Barcelona, SPAIN

## Abstract

**Background:**

Syphilis is prevalent among men who have sex with men (MSM) in China. Syphilis partner notification (PN) programs targeting MSM has been considered as one of effective strategies to prevention and control of the infection in the population. We examined willingness and preferences for PN among MSM to measure feasibility and optimize uptake.

**Methods:**

Participation in a syphilis PN program was measured using a factorial survey from both the perspective of the index patient and the partner. Respondents were recruited from April-July 2011 using convenience sampling at two sites—a MSM sexually transmitted disease (STD) clinic and a MSM community based organization (CBO). Respondents first evaluated three factorial survey vignettes to measure probability of participation and then an anonymous sociodemographic questionnaire. A two-level mixed linear model was fitted for the factorial survey analysis.

**Results:**

In 372 respondents with mean age (± SD) 28.5 (± 6.0) years, most were single (82.0%) and closeted gays (66.7%). The Internet was the most frequent place to search for sex. Few (31.2%) had legal names for casual partners, but most had instant messenger (86.5%) and mobile phone numbers (77.7%). The mean probability of participation in a syphilis PN program was 64.5% (± 32.4%) for index patients and 63.7% (± 32.6%) for partners. Referral of the partner to a private clinic or MSM CBO for follow-up decreased participation compared to the local Center for Disease Control and Prevention (CDC) or public STD clinic.

**Conclusions:**

Enhanced PN services may be feasible among MSM in South China. Internet and mobile phone PN may contact partners untraceable by traditional PN. Referral of partners to the local CDC or public STD clinic may maximize PN participation.

## Introduction

China’s syphilis epidemic continues to expand among men who have sex with men (MSM)[1, 2]. The prevalence of syphilis in MSM increased from 9.1% in 2001–2008 to 11.2% in 2009–2013 and is higher in the capital and developed coastal cities[2, 3]. The Chinese government and Ministry of Health recognized and responded to the epidemic in 2010 by launching the National Program for Prevention and Control of Syphilis in China (2010–2020)[4]. Syphilis control measures have included public awareness campaigns, free testing at voluntary counseling and testing clinics, and condom distributions[5]. Partner notification (PN) is an effective sexually transmitted disease (STD) control measure that has been underutilized in China[6]. PN is particularly useful for syphilis due to the disease’s long incubation and latency periods[7].

PN counseling for STDs is required under the National Program for Prevention and Control of Syphilis, but its implementation has been inconsistent due to lack of PN guidelines and training[4, 6]. Furthermore, traditional PN by patient, provider, or contract method can be difficult in the MSM population because MSM often find new, anonymous sex partners online and are unable to contact partners later[8]. Internet and mobile phone PN use e-mail, instant message, and short message service (SMS) to contact partners in place of traditional contact information. Internet and mobile phone PN have had high acceptability and success in high- and middle-income countries, including the United States, Australia, Peru, and South Africa[9–12].

Targeted expansion of Internet and mobile phone PN programs in China may increase the effectiveness of PN and help control the syphilis epidemic in MSM, but the feasibility of comprehensive PN programs incorporating traditional, Internet, and mobile phone PN has not been studied in China. China has a free national Internet-based SMS PN service for human immunodeficiency virus (HIV) and STD patients called EasyTell (Guangzhou Center for Disease Control and Prevention [CDC], Guangzhou) that was created by the Guangzhou CDC and a local MSM community-based organization (CBO) in 2009[13]. In 2009, 336 notifications were sent—237 (70.5%) for HIV PN and 99 (29.5%) for STD PN[13].

We examined the feasibility of a syphilis PN program that included both traditional PN and new Internet and mobile phone PN for MSM in South China. We also studied which PN program features (PN referral method, partner contact method, and partner follow-up location) optimized participation.

## Materials and Methods

### Study design

This study used cross-sectional factorial survey design[14]. Factorial surveys combine the strength of experimental research with the advantage of survey design and sampling. Factorial surveys use hypothetical vignettes to mimic real world complexities and measure respondent attitudes, judgment, and decision-making[14]. Respondents first evaluated three factorial survey vignettes and then completed an anonymous sociodemographic questionnaire. This study was approved by the institutional review boards at the University of North Carolina at Chapel Hill and the China National Center for Sexually Transmitted Diseases Control.

**Fig 1:**
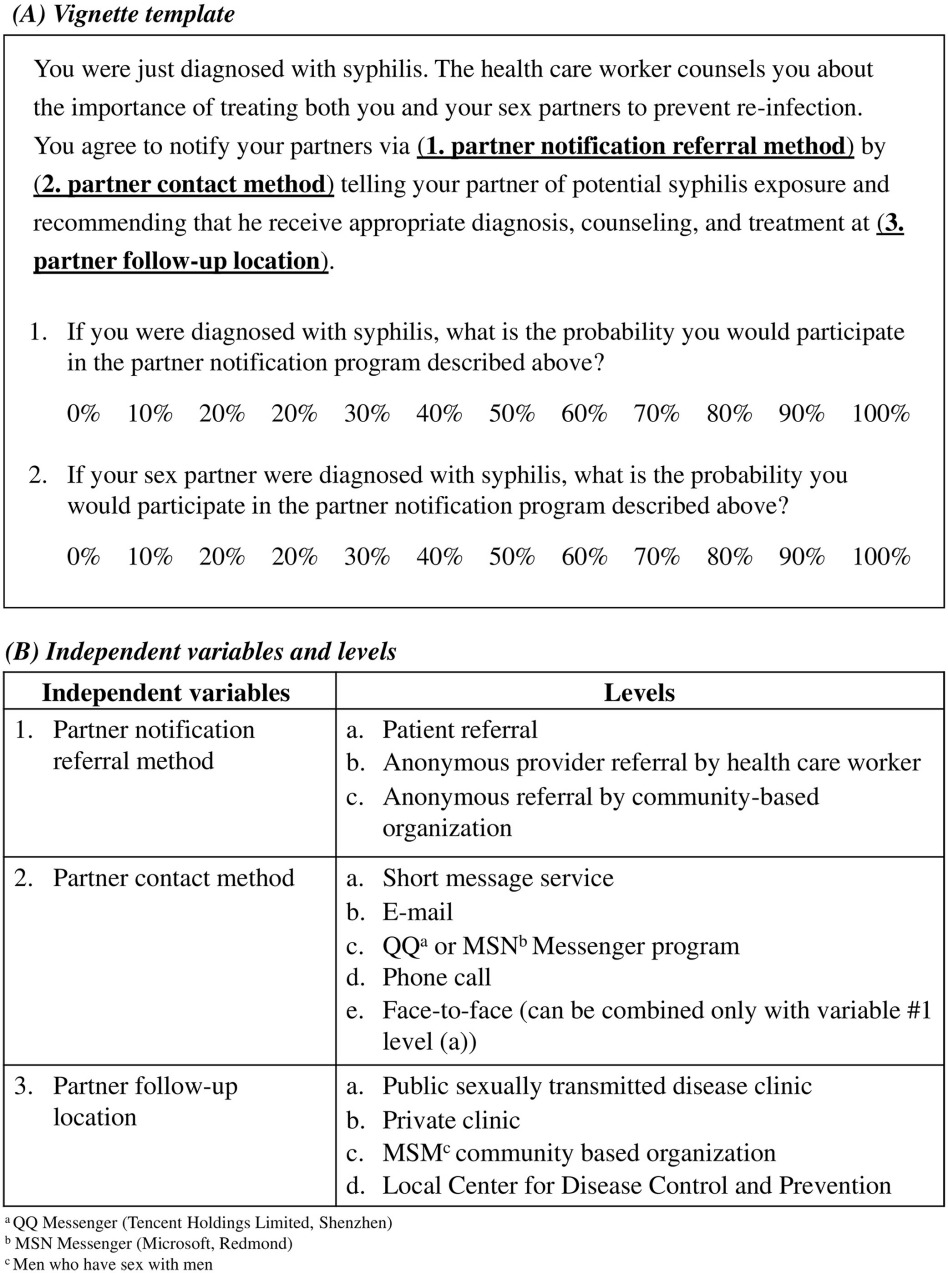
A. Factorial survey vignette template. B. Factorial survey independent variables and levels.

The factorial survey vignettes modeled hypothetical PN programs (Fig 1A) and were composed of three independent variables with three to five levels each (Fig 1B). After each vignette, respondents were asked to rate their probability of participation in the PN vignette described on a Likert-type scale of 0–100%: (1) from the perspective of the patient in the vignette and (2) from the perspective of the partner of the patient in the vignette (Fig 1A).

Vignette independent variables and levels were determined by performing a literature search and consulting MSM clinic physicians and CBOs in Guangzhou, Shenzhen, and Nanjing. Sample vignettes were distributed to MSM clinic physicians and CBO staff to review for plausibility, and the survey was pilot tested in 20 volunteer MSM in Guangzhou and Nanjing.

The sociodemographic questionnaire asked respondents for demographic, drug use, sexual behavior, STD history, and partner notification information. The questions were adapted from other MSM surveys: (1) Shanghai Men’s Study, (2) United States CDC’s National Behavioral Surveillance Survey, and (3) Fenway Community Health’s Survey On Partner Notification Activities Among MSM in Boston[15–17]. Additional questions were developed to assess for cultural barriers to PN, contact methods for casual sex partners and Internet sex partners, acceptability of Internet and mobile phone PN, and reasons for refusing PN. Barriers and reasons for refusing PN were assessed with multiple response and free text questions.

### Sample size and recruitment

Power analysis using G*3 Power 3.0.5 was performed a priori[18]. The factorial survey has three independent variables with three to five levels each comprising 52 total possible vignettes (Fig 1B). In order to detect a conservative effect size of 0.20 with α of 0.05 and power of 0.95, 839 observations were needed. Since each respondent rated 3 vignettes, a minimum of 280 respondents was needed.

Respondents were recruited from April-July 2011 using convenience sampling at two sites in Guangdong Province: (1) Foshan MSM STD Clinic in Foshan and (2) Guang Tong MSM CBO in Guangzhou. Guangdong Province in South China is the manufacturing hub of China and has one of the highest syphilis incidence and prevalence rates in China[5]. The study was advertised by instant message, word of mouth, and flyers. One research assistant (RA) at each site oversaw respondent recruitment and survey administration.

At the MSM STD Clinic, physicians referred patients interested in the study to the RA after their clinic visit. Participation was voluntary and confidential, and lack of participation did not affect the quality or cost of care. At the MSM CBO, the RA recruited respondents from visitors and members. Interested clinic and CBO staff were offered the opportunity to complete the survey at a confidential location.

Respondents had to be at least 18 years of age, be self-identified males, be able to understand Mandarin or Cantonese Chinese, be able to give informed consent, and had a history of sex (oral, anal, or both) with a male. Members and visitors of the MSM CBO in Guangzhou could potentially be patients of the MSM STD clinic in Foshan 30 kilometers away and vice versa. Both sites excluded respondents who self-reported prior participation in the survey. Verbal informed consent was obtained, and submission of the completed survey signified consent to participate. The survey was anonymous and self-administered. Respondents completed the surveys in separated and enclosed areas to ensure privacy.

### Data analysis

Data was independently double-coded with Epidata (version 3.0, Denmark) and analyzed with SPSS (SPSS, Rel. 18.0, 2009. Chicago: SPSS Inc.) and SAS (SAS, version 9.2, 2008. NC: SAS Institute Inc.). The data had a hierarchical structure by design because each respondent rated multiple vignettes. Multilevel regression analysis was used specifically to account for the hierarchical structure in the data. A two-level mixed linear model was used to fit the dataset for the factorial survey. In the model, the probability of participation in a syphilis PN program was the dependent variable. PN referral method, contact method, partner follow-up location, and study site were the independent variables. Repeated measurements within respondents were accounted for by modeling the repeated covariance matrix. The chi-square test was used to analyze sociodemographic survey data.

## Results

### Sociodemographic background

A total of 372 respondents participated, 149 in Foshan and 223 in Guangzhou. The mean age (± SD) was 28.5 (± 6.0) years, and 94.9% were Han ethnicity (Table 1). Of the total respondents, 58.2% were from Guangdong Province, 88.2% completed high school level education, and 68.0% had health insurance. Most respondents were single (82.0%), self-identified as closeted gay (66.7%), never had sex with a woman (70.4%), and never had commercial sex (94.6%) (Table 2). Testing for HIV and syphilis was common but not for other STDs. Most respondents reported negative HIV status (89.6%) and no history of STDs (67.3%). When reported, syphilis infection (14.5%) was the most frequent STD. The majority of respondents (61.3%) preferred to receive syphilis testing at the local CDC compared to other sites (χ^2^ = 2260.0, *p*<0.0001).

**Table 1:** Sociodemographic information.

	Foshan MSM^a^ Clinic	Guangzhou MSM CBO^b^	Total
	(n = 149)	(n = 223)	(n = 372)
**Mean age (years ± SD) (n = 365)**	28.8 (± 6.7)	28.3 (± 5.6)	28.5 (± 6.0)
**Ethnicity (n = 372)**			
Han	138 (92.6%)	215 (96.4%)	353 (94.9%)
Minority	11 (7.4%)	8 (3.6%)	19 (5.1%)
**Registered residence (n = 369)**			
Guangzhou	2 (1.4%)	73 (32.7%)	75 (20.3%)
Foshan	46 (31.5%)	8 (3.6%)	54 (14.6%)
Other city in Guangdong Province	31 (21.2%)	55 (24.7%)	86 (23.3%)
Outside of Guangdong Province	67 (45.9%)	87 (39.0%)	154 (41.7%)
**Education (n = 372)**			
Primary school or below	5 (3.4%)	0 (0.0%)	5 (1.3%)
Middle school	32 (21.5%)	7 (3.1%)	39 (10.5%)
High school or equivalent	54 (36.2%)	40 (17.9%)	94 (25.3%)
Junior college or equivalent	46 (30.9%)	76 (34.1%)	122 (32.8%)
College or above	12 (8.1%)	100 (44.8%)	112 (30.1%)
**Occupation (n = 372 responses, % based on cases)**			
Student	5 (3.4%)	31 (13.9%)	36 (9.7%)
Blue collar	92 (61.7%)	69 (30.9%)	161 (43.3%)
White collar	26 (17.4%)	89 (39.9%)	115 (30.9%)
Government official	4 (2.7%)	10 (4.5%)	14 (3.8%)
Freelance	12 (8.1%)	20 (9.0%)	32 (8.6%)
Sex worker	1 (0.7%)	0 (0.0%)	1 (0.3%)
Unemployed	10 (6.7%)	7 (3.1%)	17 (4.6%)
Retired	0 (0.0%)	0 (0.0%)	0 (0.0%)
Other	4 (2.0%)	8 (3.6%)	11 (3.0%)
**Monthly income in Chinese RMB**^c^ **(n = 371)**			
<1000	10 (6.7%)	31 (14.0%)	41 (11.1%)
1000–2999	86 (57.7%)	66 (29.7%)	152 (41.0%)
3000–4999	37 (24.8%)	58 (26.1%)	95 (25.6%)
≥5000	16 (10.7%)	67 (30.2%)	83 (22.4%)
**Marital status (n = 372, % based on cases)**			
Single	118 (79.2%)	187 (83.9%)	305 (82.0%)
Unmarried, cohabiting with sex partner(s)	10 (6.7%)	20 (9.0%)	30 (8.1%)
Married	19 (12.8%)	20 (9.0%)	39 (10.5%)
Divorced/separated	13 (8.7%)	15 (6.7%)	28 (7.5%)
Widowed	0 (0.0%)	0 (0.0%)	0 (0.0%)
**Health insurance status (n = 372)**			
No	55 (36.9%)	64 (28.7%)	119 (32.0%)
Yes	94 (63.1%)	159 (71.3%)	253 (68.0%)
**Illicit injection drug use (n = 371)**			
No	145 (97.3%)	222 (99.6%)	367 (98.9%)
Yes	3 (2.0%)	1 (0.4%)	4 (1.1%)

^a^men who have sex with men

^b^community based organization

^c^Renminbi

**Table 2:** Sexual behavior and sexually transmitted disease history.

	Foshan MSM Clinic	Guangzhou MSM CBO	Total
	(n = 149)	(n = 223)	(n = 372)
**Sexual orientation (n = 372)**			
Openly gay	13 (8.7%)	21 (9.4%)	34 (9.1%)
Closeted gay	99 (66.4%)	149 (66.8%)	248 (66.7%)
Openly bisexual	0 (0.0%)	2 (0.9%)	2 (0.5%)
Closeted bisexual	26 (17.4%)	46 (20.6%)	72 (19.4%)
Heterosexual	0 (0.0%)	0 (0.0%)	0 (0.0%)
Uncertain	11 (7.4%)	5 (2.2%)	16 (4.3%)
**Male sex partners in past 12 months (n = 367)**			
0	13 (9.0%)	6 (2.7%)	19 (5.2%)
1–2	74 (51.0%)	94 (42.3%)	168 (45.8%)
3–4	30 (20.7%)	64 (28.8%)	94 (25.6%)
≥5	28 (19.3%)	58 (26.1%)	86 (23.4%)
**Male sex partners in past 12 months, without condom use (n = 368)**			
0	72 (49.7%)	77 (34.5%)	149 (40.5%)
1–2	48 (33.1%)	110 (49.3%)	158 (42.9%)
3–4	12 (8.3%)	25 (11.2%)	37 (10.1%)
≥5	13 (9.0%)	11 (4.9%)	24 (6.5%)
**Anonymous male sex partners in past 12 months (n = 355)**			
0	121 (87.7%)	157 (72.4%)	278 (78.3%)
1–2	9 (6.5%)	38 (17.5%)	47 (13.2%)
3–4	3 (2.2%)	12 (5.5%)	15 (4.2%)
≥5	5 (3.6%)	10 (4.6%)	15 (4.2%)
**History of sexual contact with a woman (n = 371)**			
No	88 (59.5%)	173 (77.6%)	261 (70.4%)
Yes	60 (40.5%)	50 (22.4%)	110 (29.6%)
**History of engaging in commercial sex (n = 370)**			
No	135 (91.8%)	215 (96.4%)	350 (94.6%)
Yes	12 (8.2%)	8 (3.6%)	20 (5.4%)
**Previous HIV**^a^ **test (n = 372)**			
No	35 (23.5%)	78 (35.0%)	113 (30.4%)
Yes	114 (76.5%)	145 (65.0%)	259 (69.6%)
**HIV status (n = 259)**			
Negative	101 (88.6%)	131 (90.3%)	232 (89.6%)
Positive	4 (3.5%)	8 (5.5%)	12 (4.6%)
Unknown	9 (7.9%)	6 (4.1%)	15 (5.8%)
**Previous STD**^b^ **test (not including HIV) (n = 372)**			
No	75 (50.3%)	138 (61.9%)	213 (57.3%)
Yes	74 (49.7%)	85 (38.1%)	159 (42.7%)
STD history* **(n = 159)**			
None	49 (68.1%)	58 (68.2%)	107 (67.3%)
Syphilis	12 (16.7%)	11 (12.9%)	23 (14.5%)
Gonorrhea	4 (5.6%)	4 (4.7%)	8 (5.0%)
Chlamydia	0 (0.0%)	0 (0.0%)	0 (0.0%)
Genital herpes	2 (2.8%)	2 (2.4%)	4 (2.5%)
Genital warts	4 (5.6%)	9 (10.6%)	13 (8.2%)
Other	3 (4.2%)	1 (1.2%)	4 (2.5%)

^a^human immunodeficiency virus

^b^sexually transmitted disease

*Only answered if ever had a previous sexually transmitted disease test

The most frequent place to search for sex was on the Internet (70.1%), and 55.1% of respondents reported having had casual sex with a partner met on the Internet (Table 3). The most frequent forms of contact information to have for casual sex partners were QQ Messenger (Tencent Holdings Limited, Shenzhen) numbers (86.5%) and mobile phone numbers (77.7%). Fifty-three (14.6%) respondents reported history of being notified of potential STD or HIV exposure, most commonly via phone call (49.1%), instant message (20.8%), and face-to-face contact (15.1%).

**Table 3:** Partner seeking and notification history.

	Foshan MSM Clinic	Guangzhou MSM CBO	Total
	(n = 149)	(n = 223)	(n = 372)
**Places searched for sex in past 12 months (n = 371, % based on cases)**			
Did not look	32 (21.6%)	24 (10.8%)	56 (15.1%)
Internet	81 (54.7%)	179 (80.3%)	260 (70.1%)
MSM phone hotline	3 (2.0%)	1 (0.4%)	4 (1.1%)
Bar or dance club	17 (11.5%)	21 (9.4%)	38 (10.2%)
Gym	4 (2.7%)	12 (5.4%)	16 (4.3%)
Sauna or bathhouse	11 (7.4%)	15 (6.7%)	26 (7.0%)
Sex party	5 (3.4%)	3 (1.3%)	8 (2.2%)
Through mutual acquaintance	27 (18.2%)	43 (19.3%)	70 (18.9%)
Public area^a^	43 (29.1%)	10 (4.5%)	53 (14.3%)
MSM CBO	16 (10.8%)	13 (5.8%)	29 (7.8%)
Other	2 (1.4%)	5 (2.2%)	7 (1.9%)
**Ever had casual sex with partner met on Internet (n = 345)**			
No	83 (64.3%)	72 (33.3%)	155 (44.9%)
Yes	46 (35.7%)	144 (66.7%)	190 (55.1%)
**Contact information for casual sex partners in past 12 months (n = 260, % based on cases)**			
Legal name	22 (23.4%)	59 (35.5%)	81 (31.2%)
Mobile number	65 (69.1%)	137 (82.5%)	202 (77.7%)
Landline number	1 (1.1%)	3 (1.8%)	4 (1.5%)
QQ Messenger^b^ number	71 (75.5%)	154 (92.8%)	225 (86.5%)
MSN Messenger^c^ ID	4 (4.3%)	12 (7.2%)	16 (6.2%)
E-mail address	5 (5.3%)	18 (10.8%)	23 (8.8%)
Physical address	5 (5.3%)	27 (16.3%)	32 (12.3%)
**Ever been contacted about possible STD or HIV exposure (n = 364)**			
No	114 (80.9%)	197 (88.3%)	311 (85.4%)
Yes	27 (19.1%)	26 (11.7%)	53 (14.6%)
**Method of contact received about potential STD or HIV exposure (n = 53)**			
Face-to-face	3 (11.5%)	5 (18.5%)	8 (15.1%)
Phone call	12 (46.2%)	14 (51.9%)	26 (49.1%)
E-mail	0 (0.0%)	0 (0.0%)	0 (0.0%)
Short message service	0 (0.0%)	2 (7.4%)	2 (3.8%)
Instant message (QQ or MSN)	6 (23.1%)	5 (18.5%)	11 (20.8%)
Other	5 (19.2%)	1 (3.7%)	6 (11.3%)

^a^Public park, public restroom, metro/bus/train station, area surrounding public news bulletin boards

^b^QQ Messenger (Tencent Holdings Limited, Shenzhen)

^c^MSN Messenger (Microsoft, Redmond)

### STD partner notification history

Twenty (38.5%) of 52 respondents with history of a STD (not including HIV) reported that a health care worker provided PN counseling at the time of last STD diagnosis. Thirty-six respondents reported having a main partner at the time of diagnosis. Twenty-five (69.4%) respondents notified main partners, and 11 (30.6%) did not notify main partners. Three (21.4%) of 14 married respondents notified their spouse. Out of 11 respondents, reasons for not notifying main partners included embarrassment (n = 3), fear of relationship breakdown (n = 2), concern of the partner telling others (n = 2).

Thirty-five respondents reported having a casual partner at the time of diagnosis. Among the 35 respondents, 7 (20.0%) notified all casual partners, 5 (14.3%) notified some casual partners, and 23 (65.7%) did not notify casual partners. Out of 28 respondents, reasons for not notifying casual partners included lack of contact information (n = 8), embarrassment (n = 5), difficulty with emotional coping after diagnosis (n = 5), and lack of concern for the partner (n = 3). Other reasons for not notifying main or casual partners were fear of verbal or emotional abuse, fear of relationship breakdown, recent separation, and concern of the partner telling others.

### Willingness to participate in syphilis PN

When willingness to notify for syphilis PN was asked as a dichotomous question in the sociodemographic questionnaire, 87.2% (n = 319) of respondents were willing to self-notify sex partners, 66.7% (n = 244) were willing to notify through a physician, and 56.9% (n = 209) were willing to notify via a MSM CBO (χ^2^ = 83.380, *p*<0.0001). Out of the respondents willing to self-notify, 74.1% (n = 266) were willing to notify via face-to-face contact, 73.4% (n = 254) via SMS, 70.8% (n = 243) via instant message, 66.3% (n = 232) via phone call, and 59.9% (n = 202) via e-mail (χ^2^ = 22.181, *p*<0.0001).

Reasons for refusing to participate in syphilis PN were evaluated. Out of 47 respondents, reasons for refusing to self-notify included embarrassment (n = 17), fear of relationship breakdown (n = 15), perception of not necessary because syphilis is curable (n = 15), fear of emotional abuse (n = 13), fear of verbal abuse (n = 12), lack of partner contact information (n = 7), and fear of physical abuse (n = 4). Out of 118 respondents, reasons for refusing physician PN included desire to protect the partner’s privacy (n = 45), concern that the partner would not be notified in a sensitive manner (n = 41), perception that PN is a private matter that should not involve the physician (n = 35), and concern of breech of confidentiality (n = 28). Out of 153 respondents, reasons for refusing CBO PN included desire to protect the partner’s privacy (n = 61), concern that the partner would not be notified in a sensitive manner (n = 58), concern of breech of confidentiality (n = 54), perception that PN is a private matter that should not involve the CBO (n = 30), and concern that the CBO staff would not be trained to answer the partner’s questions (n = 25).

### Optimizing syphilis PN programs for participation

In the factorial survey analysis, the mean probability of participation in a syphilis PN program across all factorial survey vignettes was 64.5% (± 32.4%) if the respondent was the index patient and 63.7% (± 32.6%) if the respondent was the sex partner of the index patient.

Bivariate analysis of the mean probability of participation in a syphilis PN program between groups of index patients and sex partners of index patients demonstrated that PN referral method, partner contact method, partner follow-up location, and study site significantly influenced participation in a PN program (p<0.0001) (Table 4).

**Table 4:** Comparison of the mean probability of participation in a syphilis PN program (range 0–1) between infected patient and sex partner of the infected patient participant groups by levels of independent variables.

Independent variable	If the respondent was the infected patient		If the respondent was the sex partner of the infected patient	
	Mean	± SD	Mean	± SD
**PN referral method**				
Patient referral	0.6411	0.3113	0.6566	0.3068
Anonymous health care worker	0.6339	0.3276	0.6110	0.3440
Anonymous CBO	0.6594	0.3345	0.6378	0.3302
**Contact method**				
Short message service	0.6760	0.3049	0.6554	0.3184
E-mail	0.6494	0.3269	0.6479	0.3274
Instant message	0.6322	0.3373	0.6267	0.3314
Phone call	0.6238	0.3296	0.6096	0.3345
Face-to-face	0.6358	0.3075	0.6580	0.3049
**Partner follow-up location**				
Public STD clinic	0.6928	0.2997	0.6638	0.3117
Private clinic	0.5573	0.3542	0.5527	0.3479
MSM CBO	0.6276	0.3345	0.6505	0.3269
Local CDC^a^	0.6997	0.2833	0.6787	0.3040

^a^Center for Disease Control and Prevention

In the multivariate mixed linear model, only partner follow-up location significantly influenced probability of participation in a syphilis PN program for both index patient and sex partner (*p*<0.0001) (Table 5). If the respondent was the index patient, referral of the sex partner to a private clinic or MSM CBO decreased willingness to participate in the syphilis PN program compared to referral to the local CDC or public STD clinic. If the respondent was the sex partner, referral to a private clinic decreased participation.

**Table 5:** Estimates from the multiple linear regression showing the effects of the independent variables on the probability of participation in a syphilis PN program (range 0–1).

Independent variable	If the respondent was the infected patient			If the respondent was the sex partner of the infected patient		
	Coefficient (β)	SE	*P*-value	Coefficient (β)	SE	*P*-value
**PN referral method**			0.5405^‡^			0.1881^‡^
Patient referral	-0.0181	0.0243	0.4574	0.0179	0.0246	0.4651
Anonymous health care worker	-0.0262	0.0242	0.2787	-0.0268	0.0245	0.2733
Anonymous CBO	Reference	-	-	Reference	-	-
**Contact method**			0.4250^‡^			0.5442^‡^
Short message service	0.0323	0.0430	0.4534	0.0148	0.0435	0.7332
E-mail	0.0081	0.0429	0.8498	0.0095	0.0434	0.8258
Instant message	-0.0109	0.0431	0.8007	-0.0124	0.0436	0.7766
Phone call	-0.0181	0.0429	0.6726	-0.0290	0.0434	0.5042
Face-to-face	Reference	-	-	Reference	-	-
**Partner follow-up location**			<0.0001^‡^			<0.0001^‡^
Public STD clinic	-0.0063	0.0269	0.8149	-0.0141	0.0273	0.6046
Private hospital	-0.1418	0.0269	<0.0001	-0.1257	0.0272	<0.0001
MSM CBO	-0.0724	0.0270	0.0074	-0.0283	0.0273	0.2995
Local CDC	Reference	-	-	Reference	-	-

^‡^F test *P-value* for overall significance

## Discussion

PN is an effective method to control further transmission of STDs and can produce a population level decline in incidence and prevalence of infection[19]. Furthermore, PN is one of the components of the core technical strategy for the Chinese National Program for Prevention and Control of Syphilis, but implementation has been variable and needs to be further guided by research in high-risk groups, particularly in MSM[4, 6]. We surveyed MSM from a STD clinic and CBO in South China and found high reported rates of syphilis infection and casual sex with Internet partners, for which traditional contact information such as legal names and landline numbers were not commonly available. Traditional, Internet, and mobile phone PN methods were compared using factorial survey design, and MSM were found to have high willingness to participate in syphilis PN programs both as the index patient and the partner.

Our factorial survey results showed that partner follow-up location is an important factor that may influence participation in a syphilis PN program. Partner referral to the local CDC clinic or public STD clinic optimized index patient and partner participation. Whereas, partner referral to a private clinic significantly decreased index patient and partner participation. Index patients were also less likely to participate if they knew their partners would be referred to a CBO for follow-up. In China, many STD clinics affiliated with local CDCs or public dermatology-venereology hospitals are established specifically for MSM and some of these clinics involve in the outreach services to this population. Due to this historical relationship, MSM may prefer public STD clinics to private clinics[5]. The decision to seek medical care at public versus private clinics is complex and involves factors such as proximity, cost, quality, and severity of illness[20, 21]. In addition, health policy research in China has revealed public mistrust of the private health sector due to poor regulations and variable quality of care[20]. Our results may be biased as we did not recruit MSM from private clinics. However, private clinics in China are inherently difficult sites to perform scientific research due to the poor enforcement of medical licensing and regulations. Patients in rural areas also seek care at private clinics more often than patients in urban areas, and our study was performed in the third largest city in China[20]. Lastly, individual CBO PN programs were not preferred by index patients for partner referral in this study, but hybrid CBO-clinic models have been forming in China and warrant further research[22]. MSM interviewed in another study in South China valued the safe environment of the CBO but were concerned about the technical competence of STD testing at non-governmental sites and the ability to maintain anonymity at small CBOs[22]. In hybrid CBO-clinics, the CBO partners with the local CDC or public STD clinic to provide both the psychosocial counseling services of the CBO and the technical expertise and confidentiality of the local CDC or public STD clinic. Our results support the utilization of local CDC and STD clinic services for syphilis PN interventions in MSM in urban South China to optimize participation.

Self-notification had high acceptability, but respondents searched for sex online pseudonymously and often did not have traditional contact information, such as name, address, and landline number, for casual sex partners. Respondents most commonly had QQ Messenger numbers and mobile phone numbers for their sex partners. Internet and mobile phone PN using SMS, instant message, and e-mail had high acceptability as avenues for self-notification to augment PN uptake. Interestingly, our sociodemographic questionnaire revealed preferences for PN referral method and partner contact method that were not significant in the factorial survey. This discrepancy exists because the questionnaire measured willingness to notify in isolation, whereas the factorial survey was more robust and measured willingness by modeling real-world circumstances. Even so, the sociodemographic and factorial surveys both demonstrated that MSM in South China are willing to engage in PN using new electronic technologies. Similar to results in other middle- and high-income countries, including Peru, Australia, and the United States, Internet and mobile phone PN in China have the potential to contact more partners than traditional PN[9–11, 23].

We observed a low rate of PN counseling (38.5%) that magnified barriers to PN uptake. Increased PN education and counseling at the time of STD diagnosis have been shown to minimize perceived barriers and raise PN success rates[24]. Embarrassment was the most common reason for refusing self-notification and not notifying main partners in our population but has not been commonly reported in China[6]. This could be because MSM PN has not previously been studied in China, and the psychosocial issues and stigma faced by MSM index patients may be different than in the heterosexual population. Lack of contact information was the most common reason for not notifying casual partners and has been specifically reported in heterosexual syphilis and HIV index patients in China[25, 26]. Desire to protect the partner’s privacy was the most common reason for refusing physician and CBO PN, and confidentiality has been shown to be more important than the duty to warn in another marginalized population in China, pregnant syphilis patients[27]. These barriers can be lowered through individual patient counseling at the time of diagnosis, public health education, and strengthening of PN resources[28]. A study in Zambia found that individual counseling of male patients on PN at the time of diagnosis led to increased numbers of partners traced[29]. Patients in South Africa who watched an educational video presentation on STDs and PN felt more confident about notifying partners, and these patients not only notified more partners but also had more partners return to clinic[30]. PN guidelines, training, and infrastructure in China need to be expanded in order to educate patients on the importance of PN and partner treatment, in addition to providing them the resources to sensitively inform partners.

This study had several important limitations. First, we used factorial survey design and hypothetical vignettes to model real-world scenarios and decision-making. Willingness to notify has not been correlated with success due to patient and infrastructure barriers[24]. Nonetheless, this is the first study to evaluate the acceptability of PN in the Chinese MSM population, which opens the field to future research on PN interventions and uptake. We found high acceptability and probability of participation, with the predicted rate similar to the actual rate of main partner PN for respondents with history of STD. Second, mobile applications are increasingly being used by MSM to seek sex partners and was not included as a potential PN method[31]. Expansion into mobile applications would likely identify and contact even more pseudonymous partners. Third, respondents were not recruited from private clinics and may have had a bias against private clinics. Research shows that the clientele at private and public clinics intermix in China, and the factors that drive patients to seek health care at different types of clinics are intricate and multifactorial as discussed earlier[20, 21]. Fourth, although our data was collected in 2011 and remains the first survey of MSM PN in China, consideration must be given to its current applicability. Our data confirms the most recent published data on Chinese MSM sex seeking behavior using new electronic technologies and addresses an unmet need for research on PN and programmatic interventions in the MSM population[8].

In conclusion, MSM appear willing to participate in PN programs in South China, but many do not have traditional contact information for casual sex partners found anonymously online. Internet and mobile phone PN may be able to augment and contact partners untraceable by traditional PN. Self-notification had the highest acceptability, whereas partner confidentiality often preceded physician and CBO PN. In order to optimize PN participation for partner follow-up, PN programs should consider referring partners to the local CDC or public STD clinic. Health care workers need PN training as programs are implemented to provide psychosocially sensitive PN counseling and education to MSM patients and partners. Additional research is needed on the use of mobile applications as a PN method and the utilization of new health care delivery models, such as the CBO-clinic model, for PN referral. The implementation of PN varies from province to province in China, including the delegation of responsibilities and standards of practice, due to lack of operational guidelines[6]. We provided a framework for providing PN services for MSM in South China and identified areas of future development and research. The results of this study not only support the development of operational guidelines for MSM PN in China but also demonstrate a need for expanded PN implementation research in China.
